# On the Construct of Subjective Risk Intelligence and Its Relationships with Personality, Emotional Intelligence and Coping Strategies: A Comparison between Adolescents and Adults

**DOI:** 10.3390/ejihpe14060102

**Published:** 2024-06-01

**Authors:** Maria Guarnera, Rita Zarbo, Stefania Lucia Buccheri, Paola Magnano

**Affiliations:** Department of Human and Social Sciences, Kore University, Cittadella Universitaria, Plesso di Psicologia, 94100 Enna, Italy; maria.guarnera@unikore.it (M.G.); rita.zarbo@unikore.it (R.Z.); paola.magnano@unikore.it (P.M.)

**Keywords:** Big Five, coping, emotional intelligence, risk intelligence, adolescents, adults, personality

## Abstract

The complexity of today’s scenario has made it necessary to investigate the need for individuals to make choices that entail increasing exposure to risk and uncertainty. Among the individual resources that could help people to cope with situations of uncertainty, the new construct of subjective risk intelligence (SRI), known as a person’s ability to effectively weigh the pros and cons of a decision in situations where not all the outcomes are foreseen, would seem to play a prominent role. Considering that personality and coping strategies have been shown to be significantly related in previous research, the present study investigates the relationships between subjective risk intelligence, emotional intelligence, personality traits and coping strategies in both adults and adolescents. This cross-sectional study was conducted on 1390 Italian people, divided into two subsamples of 641 adolescents and 749 adults. The results showed that SRI mediated the roles that personality traits and emotional intelligence have in coping strategies differently in the adult sample, in which the mediating role of SRI was found for avoidance coping, and in the adolescent sample, in which SRI influenced all of the antecedents analysed in the study for almost all of the identified coping strategies. In light of these findings, subjective risk intelligence could be activated to deal with uncertain and risky situations, influencing the choice of effective or ineffective strategies in both adults and adolescents.

## 1. Introduction

According to Beck [1,2], the prominent characteristic of today’s society—defined by the author himself as a “risk society”—is the omnipresence of risks. This omnipresence has led to growing exposure to new, unpredictable, unknown and unprecedented risks that require a necessary reconfiguration not only of the subjective perception of risk but also of the inter-subjective communication of risk and the social experience of living in a risky environment.

Moreover, coping with such an uncertain environment represents a transgenerational challenge. Adults who grew up in eras characterised by greater linearity and certainty now are requested to reconfigure their traditional approach to life circumstances. Adolescents are called to face not only the challenges typical of their life stage but also the disorientation dictated by the complexity of and sudden changes in the surrounding social and economic context. Individual and contextual resources are involved in such processes, and a number of studies have attempted to explain the relationship between certain personality characteristics and the coping strategies implemented by individuals to cope with stressful situations. The results of these investigations are controversial to the extent that variables, such as stress exposure, stress reactivity, and situational demands, linked to the context or the individual, may play an important role in this relationship [3]. Similar results of studies highlighting both direct [4] and indirect effects, mediated, for example, by personal variables such as self-efficacy [5] or contextual variables such as social support [6], have shed light on the possible relationships between emotional intelligence (EI) and coping strategies. Among the individual resources, a new construct has been recently proposed by Craparo et al. [7], which is defined as subjective risk intelligence (SRI), “the capacity of a person to effectively assess the pros and cons of a decision in situations in which not all outcomes are totally expected” [7] (p. 968). It consists of four sub-dimensions: (1) imaginative capability, an individual’s ability to produce new and original ideas in relation to the objective; (2) problem-solving self-efficacy, their belief to handle situations and make decisions effectively; (3) stress management, their capacity to modulate adaptively emotional responses in stressful situations; (4) a positive attitude towards uncertainty, the attribution of positive meaning to uncertainty.

This study aims to investigate how personality, EI and the potential mediator effect of SRI could act with respect to more or less adaptive coping responses for both adults and adolescents. In the theoretical part of the article, we will present the literature review on the constructs included in the empirical research, focusing on the dynamic interaction between them; the rationale of the study, derived from the previous part, will explain the underlying reasoning behind the model tested; then, the empirical research will test the relationships hypothesised; the discussion of the results in light of the existent literature will lead to the conclusions and suggestions for further research and practice.

### 1.1. SRI, Personality and EI

As the construct of subjective risk intelligence (SRI) has been recently operationalised and the literature including this new conceptualisation is limited, we have focused the literature review on the relationships between SRI, personality and EI according to its four constitutive dimensions.

Imaginative capability is a kind of creativity that can make people exceed their past experience and create meaningful and complete conceptual possibilities by organising fragmented situations [8,9]. Over the years, a large number of studies have investigated personality correlates of creativity [10]. Numerous researchers—using the Big Five model—have found that creativity is linked to high openness, low agreeableness, low conscientiousness and high neuroticism [11,12]. The same patterns of relationships have been shown by studies conducted with adolescents. In a review of studies concerning the development of creativity during adolescence, van der Zanden, Meijer and Beghetto [13] found that in some studies introversion was positively related to creativity. Openness to experience can be considered a key correlate of creativity, though [14]. The findings related to the influence of neuroticism and extraversion on creative personality suggest that affect-related processes may play an important role in creativity [15], but with respect to the relationship between EI and creativity, the studies provided mixed findings. Sànchez-Ruiz et al. [15], using the Trait EI model, found correlations between EI and creative personality but not with divergent thinking. Also, in adolescents, controversial results were found in several studies [16,17].

Regarding the second component of SRI, problem-solving self-efficacy, the results of several studies [18,19,20] indicate that personality traits and general self-efficacy are correlated. Zakiei, Vafapoor, Alikhani, Farnia and Radmher [21] found that the trait of higher neuroticism is accompanied by lower general self-efficacy. Additionally, the more the features of extraversion, agreeableness, conscientiousness and openness to experience are increased, the higher general self-efficacy will be. According to some studies on the relationship between EI and self-efficacy, highly emotionally intelligent individuals, feeling confident in their ability to adapt to new conditions and capable of dealing with pressure and regulating stress, are expected to have a high sense of personal efficacy [22,23,24,25].

On the third dimension of SRI, stress management, several studies linked the likelihood of experiencing stressful situations and appraisal of an event as stressful to personality traits [26,27]. Individuals high in neuroticism experience more stressful events, whereas individuals high in extraversion experience both more stressful and more pleasurable events [28,29]. According to several studies, individuals with higher EI cope better with the emotional demands of stressful encounters because they are able to accurately perceive their emotions and can effectively regulate their mood states [30,31].

The fourth dimension of SRI, a positive attitude toward uncertainty, is comparable to the tolerance of uncertainty, a personality trait underlying the ability to accept uncertainty and act confidently in unstable life situations [32,33]. Uncertainty tolerance can be considered an individual trait, the psychological attitude of a person or an emotional reaction to the unknown [34]. Borracci, Ciambrone and Arribalzaga [35], in their study, found that conscientiousness, extraversion and openness were associated with lower tolerance for complexity, risk and ambiguity; conversely, the trait of agreeableness was related to a higher tolerance toward risk. The results of a study conducted with adolescents [33] revealed that uncertainty tolerance as a personality trait is manifested through sensitivity, flexibility and openness to new experiences, confidence and activity in familiar and unfamiliar situations. With regard to the relationship between EI and tolerance toward uncertainty, Vahedi and Fatemi [36] found no correlations between these two constructs.

### 1.2. Personality, EI and Coping in Adults and Adolescents

The construct of coping refers to the way in which people try to manage traumatic events or stressful everyday situations. Coping strategies are used to regulate disturbing emotions and to generate solutions to manage and resolve the cause of stress [37]. Personality and coping play both independent and interactive roles in influencing physical and mental health; therefore, it is not surprising that the research on the relationship between coping and personality is very broad.

As part of research that has studied the relationship between the personality dimensions measured by the Big Five and coping, several type of studies, cross-sectional, longitudinal and meta-analytic, tend to confirm that neuroticism is associated with passive and maladaptive strategies [29] or disengagement coping [38]; extraversion and conscientiousness are strongly related to active strategies [38,39].

The relationships between the five-factor traits and emotion-focused coping also suggest the importance of distinguishing between the types of emotion-focused coping [3].

Fickovà [40], examining the coping behaviour of adolescents in relation to personality dimensions, showed that neuroticism facilitates a preference for using maladaptive or ineffective strategies in coping with stress. Extraversion has a closer relationship to searching for social support and positive reinterpretation. Openness and agreeableness have a weak relationship with coping strategies, while conscientiousness seems to be the strongest predictor of coping behaviour: people high in conscientiousness have a tendency to prefer strategies focused on the problem itself, and individuals with a low conscientiousness score prefer maladaptive strategies. A meta-analysis by Connor-Smith and Flachsbart [3] showed that personality better predicted coping in younger samples, assuming on the one hand that responses to stress are driven more strongly by temperament in younger individuals and, on the other hand, that age-related personality changes [41] may also have an impact.

A relevant body of empirical evidence suggests that EI correlates robustly with coping, particularly rational/problem-focused coping [42,43]. In one study, Noorbakhsh et al. [44] showed that EI was positively associated with problem-focused and positive-emotion-focused coping strategies and negatively associated with negative-emotion-focused coping strategies.

The relationship between EI and coping is unclear regarding adolescence, as empirical studies report very different results. The study conducted by Mohammadi et al. [45] showed that students with higher EI use both effective and non-effective coping strategies while encountering stressful situations, though they use effective coping strategies more than non-effective ones. MacCann, Fogarty, Zeidner and Roberts [46] showed that EI was positively related to academic problem-focused coping, negatively related to emotion-focused coping and not related to avoidant coping. However, the strongest relationship between EI and coping was for problem-focused coping.

### 1.3. SRI and Coping

According to Rohrmann [47], different risk-taking or risk avoidance tendencies result from ways of feeling, thinking and behaving that can be learned or socially developed. This, in turn, can influence our sense of agency, coping, self-efficacy and decision-making in adaptive or maladaptive ways, depending on the flexibility of individuals.

Important links are shown between SRI and coping strategies. As regards the four dimensions of the construct, imaginative capability had stronger relationships with both perceived problem-solving self-efficacy and problem-focused coping [7,48]. Furthermore, adaptive coping strategies seem to be associated with dimensions similar to imaginative capacity, such as creativity. For example, it has been shown that in adolescence, a period characterised by a riskier attitude and behaviour, not all risk-taking is negative but rather can give rise to socially approved behaviour, such as creativity [49,50].

Regarding the ability to manage adaptively with stress, some studies, starting from the consideration that some high-risk individuals do not show clear signs of psychological distress, have shown that resilient adolescents also exhibit high levels of problem-solving-centred coping strategies [51].

With respect to more or less positive attitudes and tolerance towards uncertainty, the advent of the COVID-19 pandemic has provided fertile ground for the study of these dimensions and the effects they may have on mental health. Rettie and Daniels [52], for example, investigated some factors that influenced mental health during the pandemic, highlighting the mediating role of maladaptive coping responses in the predictive relationship between uncertainty intolerance and psychological distress. Moreover, even before the pandemic, less recent studies highlighted the effects of uncertainty on coping. An experiment with 180 undergraduate students, for example, showed that participants in emotional states of uncertainty were more oriented towards problem-focused coping than participants in states of emotional certainty, who engaged in more emotion-focused coping [53].

Finally, regarding the dimension of problem-solving self-efficacy, the role of the resources that individuals adopt to cope with stressful situations has long been an area of great interest for psychology [54]. In this area of study, high self-efficacy has been associated with active or adaptive coping and low self-efficacy with passive or maladaptive coping [55,56,57].

### 1.4. The Rationale of the Study

Stressing events are recurrent in our life span; they are often associated with transition periods and changes in significant contexts during life, representing development and growth opportunities if adequately faced. Among personal resources, SRI could be useful for dealing with challenging situations; SRI has been found to be related to the Big Five personality traits and the trait of EI [7]. Moreover, coping strategies enable, on the one hand, a range of adaptive responses that achieve their intended purpose, and on the other, maladaptive responses that are not finalised to overcome the perceived threat [58]. Therefore, we hypothesise that SRI could strengthen coping strategies in achieving goals. This could affect positive coping strategies, reinforcing their adaptive effect on a troubled individual’s relationship with a stressful environment [59] and mitigating the maladaptive effect of negative coping strategies in stressful situations, which impede adjustment [60]. Then, considering that SRI is involved in facing one’s psychological challenges, especially in uncertain and risky conditions, and that personality characteristics, including EI, may play a role in coping strategies [61], we hypothesise that SRI is a mediator of this relationship, playing a role in individuals’ choices of coping strategies. As highlighted in the literature review, given the developmental nature of these complex patterns of relationships, it is reasonable to expect that several differences could occur among adolescents and adults due to the development of their cognitive and emotional dimensions.

**Figure 1:**
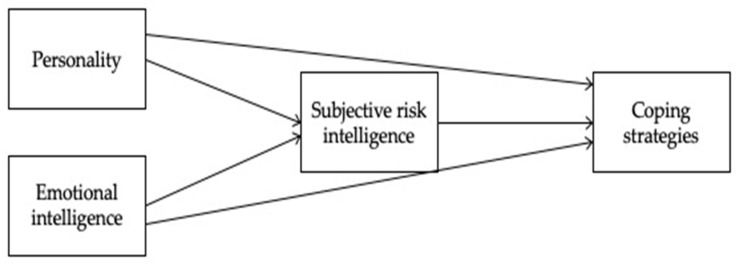
The conceptual model.

Given the extent of support among these links, Figure 1 serves as a conceptual model in our study on the relationships between personality, EI, SRI and coping strategies.

We assume then that the model produces the following answers:

1. Personality traits and EI are antecedents of SRI because they can promote or impede an intelligent risk perception and evaluation, which give the necessary push to action.
2. Individuals assess their ability to cope with stressful situations effectively or ineffectively, and this may be mediated by the person’s resources and then by a successful assessment. SRI drives the person to perform the action.
3. This pattern is applicable to adolescents and adults, even though some differences are expected due to the different cognitive and emotional development in adolescence with respect to the adult stage.

### 1.5. Aims

Following the rationale of the study, we hypothesise that:

1. Adolescents and adults have differences in SR, due to the different degrees of development of psychological resources.
2. Personality traits and EI are antecedents of coping strategies.
3. SRI plays a mediational role in the relationship at point 2.
4. The mediational role of SRI could be different in adolescents and adults due to point 1.

## 2. Materials and Methods

An a priori power analysis was conducted using G*Power software, version 3.1 [62,63], to evaluate the minimum sample size to predict coping strategies with six predictor variables. The parameters indicated in the literature were maintained to carry out this analysis: a medium effect size of 0.15 (Effect size f^2^ = 0.15) with alpha = 0.05 and minimum Power (1 − beta) = 0.95 [64]. The analysis revealed that a minimum total sample size of 146 participants was necessary.

The research project was conducted in accordance with the Declaration of Helsinki (https://www.wma.net/policies-post/wma-declaration-of-helsinki-ethical-principles-for-medical-research-involving-human-subjects/ accessed on 9 January 2023) and received the approval of the Internal Review Board of the university involved (UKE-IRBPSY-10.15.01.). This study is part of a larger research project, aimed at validating the construct of subjective risk intelligence and its related scale in adolescents and adults; other results, obtained from the same samples, have been already published [7,48].

### 2.1. Participants

Sample 1 was composed of 641 Italian adolescents (males = 278; females = 363), aged between 13 and 18 (M = 15.78; SD = 1.48), attending the different classes of various high school courses of study (20.6% first year; 20.1% second year; 20.3% third year; 23.2% fourth year; 15.8% fifth year) and recruited on a voluntary basis. The age range followed the statistical and juridical cut-off of the age of maturity, which in Italy is 18 years old. The schools were recruited through convenience sampling; the administration of the survey took place in person during school activities. The students filled out the questionnaire according to these instructions: “The following questionnaire aims to analyse behaviors and feelings in facing situations of uncertainty or difficulty. You will find below a series of statements describing common situations or behaviors, to which we ask you to indicate how much they correspond to what you do or feel, using the scale provided. There are no right or wrong answers; we ask that you answer all questions as sincerely as possible […]”. The informed consent of the students’ parents was managed by the schools. The students filled out the questionnaire during lesson time with the consent of their teachers.

Sample 2 was composed of 749 Italian adults (males = 275; females = 474), aged between 19 and 79 (M = 30.93; SD = 12.45). They were recruited through convenience sampling from the general population. More than half of them had graduated high school (60.2%), 11.9% had a middle school diploma and the others had a university degree (11.6% bachelor’s degree, 12.1% five-year university degree, 4% post-graduate degree). The participants completed the questionnaires according to the following instructions: “The following questionnaire aims to analyse behaviours and feelings in facing uncertainty or difficulty. You will find a series of statements that describe common situations or behaviours, to which we ask you to indicate on the expected range, how much they correspond to what you do, or you feel […]”. Their participation was completely voluntary. We administered the tests individually and anonymously.

### 2.2. Measures

**Big Five Inventory.** The Big Five Inventory [65,66] is composed of 10 items assessing the Big Five dimensions on a 5-point Likert scale from 1 (*strongly disagree*) to 5 (*strongly agree*). The sample items included “I see myself as a person who is outgoing, sociable” and “I see myself as a person who gets nervous easily”. Cronbach’s alpha values for the five factors ranged from 0.71 to 0.77.

**Subjective Risk Intelligence Scale**. The Subjective Risk Intelligence Scale (SRIS) [7] is a self-report scale composed of 21 items with a 5-point Likert-type scale from 1 (*totally disagree*) to 5 (*totally agree*). The SRIS was created and validated for the Italian population and is composed of twenty-one items that describe behaviours or moods. The items are grouped into four dimensions: (1) emotional stress management (3 items, sample item: “My mental state affects my work/school performance”) measures the capacity to modulate emotional responses in stressful situations; (2) a positive attitude toward uncertainty (5 items, sample item: “I am afraid of change”) refers to the ability to perceive uncertainty as an opportunity rather than a threat, attributing positive significance to it; (3) imaginative capability (7 items, sample item: “With a new project, I look for non-traditional approaches”) refers to the generation of novel and potentially useful ideas, emphasising the attributes of initiative-taking and originality, with this dimension including an individual’s ability to explore the unknown; (4) problem-solving self-efficacy (6 items, sample item: “I feel able to do all right, even in unexpected circumstances”) comprises both self-confidence and belief in one’s capacity to handle situations, including the ability to make decisions. For the study’s purposes, we used the SRIS total score (Cronbach’s alpha and McDonalds’ omega = 0.89).

The Subjective Risk Intelligence Scale for Adolescents (SRIS-A) [48] was used to measure SRI in sample 1. It is derived from the adult version and is composed of 19 items grouped into the same four dimensions (3 items for emotional stress management, 5 items for a positive attitude toward uncertainty, 6 items for imaginative capability and 5 items for problem-solving self-efficacy). For the study’s purposes, we used the SRIS total score (Cronbach’s alpha and McDonald’s omega = 0.83).

**The Self-Report Emotional Intelligence Test.** The Self-Report Emotional Intelligence Test (SREIT) [67], the Italian adaptation [68], is composed of 33 items, answered on a 5-point Likert scale from 1 (completely disagree) to 5 (completely agree). The EI total score was calculated for 30 items, excluding items 5, 28 and 33, as indicated in the Italian validation study [68]. The scale assesses how effectively respondents typically identify, understand, regulate and harness emotions in themselves and others. Cronbach’s alpha and McDonald’s omega, calculated on the samples of the study, were good (sample 1: Cronbach’s alpha and McDonald’s omega = 0.85; sample 2: Cronbach’s alpha and McDonald’s omega = 0.92). A sample item is “I know why my emotions change”.

**Coping Orientation to Problems Experienced.** The Coping Orientation to Problems Experienced test (COPE) [37,61] is a self-report scale composed of 60 items that evaluates the use of skills and strategies adopted to face stressful and difficult events in adults. For the purpose of this study, the five dimensions were grouped into two typical coping strategies [69]: self-directed coping (having a positive attitude and problem solving, 24 items in total) and self-avoidant coping (using avoidance strategies and having a transcendent orientation, 24 items in total). The dimension of social support was not considered in this study to align the dimensions with the adolescent scale. For the self-directed coping sample item “I make a plan of action”, Cronbach’s alpha was 0.79, and McDonald’s omega was 0.80; for the self-avoidant coping sample item “I give up the attempt to get what I want”, Cronbach’s alpha was 0.86, and McDonald’s omega was 0.87.

**Coping Inventory for Stressful Situations—Adolescent version.** The Italian adaptation of Coping Inventory for Stressful Situations (CISS), the adolescent version [70,71], was used to measure coping strategies in adolescents. It is composed of 48 items on a 5-point Likert scale ranging from “not at all” to “very much” that evaluate three types of coping strategies, task-oriented (16 items, sample item: “I try to understand the situation”; Cronbach’s alpha = 0.83; McDonald’s omega = 0.84), emotion-oriented (16 items, sample item: “I focus on my inadequacies”; Cronbach’s alpha = 0.84; McDonald’s omega = 0.85) and avoidance-oriented (16 items, sample item: “I buy something”; Cronbach’s alpha = 0.84; McDonald’s omega = 0.84).

In the present study, following the literature review, we considered both self-directed and task-oriented strategies as adaptive or effective strategies; on the other hand, self-avoidant, avoidance-oriented and emotion-oriented coping (as the items included in the CISS are referred to as negative emotions) were considered maladaptive or ineffective strategies. Even though the emotion-oriented strategies are not included in the COPE for adults, we took them into account in the data analysis due to their relevance to adolescents.

### 2.3. Data Analysis

We analysed the survey data using path analysis in jamovi 2.3.21 [72] to test the mediation model shown in Figure 1. The indirect effect was tested using a bootstrap estimation approach with 5000 samples and the 95% bias-corrected percentile method [73].

We also implemented other well-known analytical tools, such as the t-test for independent samples and its effect size and correlations, using SPSS 25.0 [74].

## 3. Results

### 3.1. Descriptive Statistics and Correlations

Table 1 presents the means, standard deviation and correlations between the variables included in the study in the two different subsamples (sample 1: adolescents; sample 2: adults). As shown in Table 1, in sample 1, emotion-oriented coping is not correlated with any of the personality traits, EI or SRI; task-oriented coping has similar patterns in the adolescent and adult samples: task-oriented coping and self-directed coping—which are overlapping strategies—are positively associated, in both samples, with conscientiousness, openness, EI and SRI; in older participants, task-oriented coping is also positively associated with emotional stability. Avoidance coping is related to EI in both samples but in a different way: the relationship has a positive direction in adolescents and a negative direction in adults; in younger participants, avoidance coping is also positively associated with extraversion, in adults, it is negatively correlated with conscientiousness, emotional stability and SRI. The variables were also checked for their normal distribution, computing skewness and kurtosis and considering all the dimensions with values in the range of −1/+1 normally distributed. The responses were approximately normally distributed, with the skewness ranging from −0.37 to 0.18 in sample 1 and from −0.39 to 0.85 in sample 2 and the kurtosis values ranging from −0.60 to 0.29 in sample 1 and from −0.68 to 0.55 in sample 2.

### 3.2. Differences in SRI between Adolescents and Adults

With the aim of comparing the levels of SRI and its components in the two samples of adolescents and adults, we used Student’s *t*-test (*p* < 0.05) for independent samples and Cohen’s *d* to calculate the effect size. The results are reported in Table 2. The differences between adolescents and adults are all statistically significant, with a medium effect size for both the single dimensions and the total score.

### 3.3. Path Analysis and Mediation Model

To verify the research hypotheses, we tested our conceptual model (see Figure 1) on the two subsamples. Even though the two models tested—one on adolescents and one on adults—are conceptually identical but use different or slightly different measures and indicators, we cannot use statistical indexes for their comparison, but we will explain the results through a theoretical reflection.

The path analysis and the mediation model tested on sample 1 (adolescents) show excellent fit indexes, CFI = 1.00, so the data are adequate to explain the hypothesised model; the R^2^ values are reported in Table 3. The mediation model shows the following results: the path from agreeableness to SRI is significant (β = –0.08, *p* = 0.02), as is the path from SRI to task-oriented coping (β = 0.25, *p* < 0.001). Moreover, the indirect effect (IE) is also significant (as reported in Table 4), confirming the full mediation of SRI of the relationship between agreeableness and task-oriented coping, as a direct effect of agreeableness on task-oriented coping was not found. Similarly, the path from SRI to emotion-oriented coping is significant (β = –0.48, *p* < 0.001), as well as the path from agreeableness to emotion-oriented coping (β = –0.07, *p* = 0.03); as a direct effect of agreeableness on emotion-oriented coping is confirmed, we found a partial mediation of SRI of the relationship between agreeableness and emotion-oriented coping. Then, the path from conscientiousness to SRI is significant (β = 0.12, *p* < 0.001), as well as the path from risk intelligence to task-oriented coping (previously reported), emotion-oriented coping (previously reported) and avoidance-oriented coping (β = –0.12, *p* = 0.004). The indirect effects are also significant, as well as the direct effects (Table 4), confirming partial mediation. Third, the path from emotional stability to SRI is significant (β = 0.37, *p* < 0.001), as well as the paths from SRI to the three coping strategies, as previously reported. The indirect effects, reported in Table 4, are all significant, while a direct effect is not found in the relationship between emotional stability and avoidance-oriented coping, confirming full mediation. Moreover, the path from extraversion to SRI is significant (β = 0.10, *p* = 0.01), as well as the paths from SRI to the three coping strategies, as previously reported. The indirect and the direct effects reported in Table 4 are all significant, confirming partial mediation. Furthermore, the path from openness to SRI is significant (β = 0.09, *p* = 0.01), as well as the paths from task-oriented and emotion-oriented coping, as previously reported. The indirect effects reported in Table 4 are all significant, while the direct ones are not significant, confirming full mediation. Lastly, the path from EI to SRI is significant (β = 0.22, *p* < 0.001), as well as the paths from SRI to the three coping strategies, as previously reported. The indirect and direct effects reported in Table 4 are all significant, confirming partial mediation.

The path analysis and the mediation model tested on sample 2 (adults) show excellent fit indexes, CFI = 1.00, so the data are adequate to explain the hypothesised model; the R^2^ values are reported in Table 5. The mediation model shows the following results: the path from agreeableness to SRI is not significant (β = –0.009, *p* = 0.78); nor is the path from SRI to self-directed coping (β = –0.04, *p* = 0.36). Therefore, agreeableness has only a direct effect on self-directed coping, and SRI does not play any mediating role either in this relationship or in the relationship between agreeableness and avoidance coping, nor is any direct effect of agreeableness on avoidance found (see Table 6 for direct and indirect effects). The same path is shown in the relationship between openness and coping strategies: only a direct effect is found between openness and self-directed coping, with no mediating role of SRI and no direct effect on avoidance coping. Third, the path from conscientiousness to SRI is significant (β = 0.12, *p* < 0.001), as well as the path from SRI to avoidance coping (β = –0.33, *p* < 0.001). The indirect effect is also significant, as well as the direct effect (Table 6), confirming partial mediation. Then, the path from emotional stability to SRI is significant (β = 0.35, *p* < 0.001), as well as the path from SRI to avoidance coping, as previously reported. The indirect effect, reported in Table 6, is significant, while a direct effect is not found in the relationship between emotional stability and avoidance coping, confirming full mediation. Moreover, as the path from extraversion to SRI is significant (β = 0.06, *p* = 0.023), as well as the path from SRI to avoidance coping, as previously reported, the indirect effect is significant. Lastly, the path from EI to SRI is significant (β = 0.49, *p* < 0.001), as well as the path from SRI to avoidance coping, as previously reported. The indirect effect reported in Table 6 is significant, while the direct effect is not significant, confirming full mediation.

## 4. Discussion

This study presents some relevant results. As we hypothesised, SRI can be considered a mediator between personality and EI on the one hand and coping strategies on the other, both in adolescents and in adults. For greater clarity of the exposition, detailed discussions of the results will be provided here first with reference to the adolescent sample and then with reference to the adult sample.

In the adolescent sample, the mediating role of SRI was found for all the antecedents analysed in the study for almost all of the coping strategies detected. In detail, SRI is a partial mediator of the following relationships: EI, conscientiousness and extraversion with all the coping strategies (task-oriented, emotion-oriented, avoidance-oriented); agreeableness with emotion-oriented coping; emotional stability with task-oriented and emotion-oriented coping. Moreover, SRI plays a full mediational role in the following relationships: agreeableness with task-oriented coping; emotional stability with avoidance-oriented coping; openness with task-oriented and emotion-oriented coping. Finally, SRI has no mediational role in the relationships between agreeableness and openness with avoidance-oriented coping.

In detail, regarding the role of SRI in the partial mediation between EI and coping strategies, the results can be compared to the literature on EI: people high in EI are thought to be better equipped to deal with stressful events. Their ability to accurately perceive, understand and manage their own and other peoples’ emotions should result in better coping skills [75]. In our model, EI has a positive relationship with SRI; the latter has a positive relationship with task-oriented coping and a negative relationship with emotion-oriented and avoidance-oriented coping; the indirect effects of SRI on coping strategies have the same direction as the single paths. Therefore, EI, characterised by the appraisal and expression of emotion in oneself and others, regulation of emotion in oneself and others and utilisation of emotions in solving problems, enhances SRI as a personal resource that integrates emotions and cognitions in approaching risky and uncertain situations. It is interesting to note that EI, without the mediation of SRI, has a positive relationship with emotion- and avoidance-focused coping. Probably, in adolescents, EI, improving the expression of both positive and negative emotions, without the mediation of SRI, would favour maladaptive (emotional and avoidance) coping. So, SRI can be considered a protective factor in the activation of emotional coping—which is mostly centred on self-criticism and negative emotional states—and of avoidance coping—which, according to the scale used, is focused mostly on venting emotions to others.

Extraversion, grounded in an approach temperament, involves sensitivity to reward, positive emotions, sociability, assertiveness and high energy [76,77,78]. Strong approach tendencies and assertiveness should provide the energy required to initiate and persist in problem solving [79,80]; a positive affect should facilitate cognitive restructuring and an orientation toward others and access to a social network should facilitate social support coping. According to our results, extraversion has a positive relationship with SRI; the latter, in its mediational role between extraversion and coping strategies, positively affects task-oriented coping and negatively affects emotion- and avoidance-oriented coping. Extraversion, features of which are energy and dynamism, strengthens SRI, which, in turn, allows individuals to deal with uncertain situations with an effective attitude; thus, adolescents higher in SRI tend to use active coping strategies more frequently than passive coping strategies. However, interestingly, extroverted adolescents, without the mediation of SRI, tend to use more avoidance than active coping. This pattern can be explained by the characteristic of avoidance coping in the scale used, which is essentially social distraction. Therefore, SRI could act as a protective factor that leads energy and dynamism toward a more effective coping strategy, rather than social distraction.

The dimension of emotional stability is debated in the literature in relation to its opposite, neuroticism [81], and is grounded in an avoidance temperament, reflecting tendencies to experience fear, sadness, distress and physiological arousal [77,78,82]. Given this vulnerability to distress, neuroticism should lead to emotion-focused coping and disengagement from threats [79]. Regarding our results, emotional stability has a positive relationship with SRI; SRI, as mediator between emotional stability and coping strategies, has a positive indirect effect on task-oriented coping strategies and a negative one on emotion- and avoidance-oriented strategies. Emotional stability, characterised by effective emotional management, enhances SRI as a resource that comprises emotional stress management [7,48] in risky and uncertain situations, so that adolescents with higher risk intelligence, being less vulnerable to emotional stress, are more likely use active coping strategies than ineffective ones. Similarly to what was discussed for extraversion, but surprisingly, emotionally stable adolescents, without the mediation of SRI, tend to use task-oriented coping less; probably, some developmental features could affect this relationship, i.e., some scholars have demonstrated that cognitive and emotional immaturity, characterised by less awareness of the positive expected outcomes, could inhibit the use of task-oriented coping strategies [83]

Openness to experience involves being imaginative, creative, curious, flexible, attuned to inner feelings and inclined toward new activities and ideas [77,84]. These tendencies may facilitate engagement coping strategies that require considering new perspectives, such as cognitive restructuring and problem solving, but may also facilitate the use of disengagement strategies, such as wishful thinking [85,86]. In our model, openness has a positive relationship with SRI, which, playing a mediational role between openness and coping, positively affects task-oriented coping, negatively affects emotion-oriented coping and has no effect on avoidance-oriented coping. Openness, characterised by curiosity towards novelty, improves SRI as a resource that enables individuals to face risk and uncertainty with a positive attitude and imaginative capability [7,48], inducing active coping strategies.

Agreeableness involves high trust, altruism, compliance and concern for others [76,77]. Agreeable people become less angry over others’ transgressions than less agreeable people [87] and are able to inhibit their negative feelings [88]. Agreeableness is often characterised as being broadly concerned with the maintenance of relationships [89]. Accordingly, agreeableness plays a limited role in the stress process, so it should be unrelated to most engagement and disengagement strategies [3]. In our model, agreeableness has a negative relationship with SRI; the indirect effect of agreeableness on task-oriented coping, through the mediation of SRI, is negative; conversely, the indirect effect of agreeableness on emotion-oriented coping, through the mediation of SRI, is positive, while no effect is found on avoidance-oriented coping. So, in adolescents, agreeableness, that is, an orientation towards others, implying trust, empathy and cooperation, distracts from the use of personal resources, as SRI, representing a risk factor that limits the activation of effective coping, enhances emotion-oriented strategies. On the contrary, without SRI’s mediation, agreeableness has no effect on active coping or avoidance, limiting emotional coping.

Finally, conscientiousness implies persistence, self-discipline, organisation, achievement orientation and a deliberative approach [76,77]. The strong attention regulation capacity underpinning conscientiousness [90] should predict success in cognitive restructuring, which requires a capacity to disengage from powerful negative thoughts. The results concerning conscientiousness show that the path from conscientiousness to SRI is positive: the mediational role played by SRI, on the one hand, strengthens the relationship between conscientiousness and task-oriented coping, and on the other, weakens the relationship between conscientiousness and emotion- and avoidance-oriented coping. Conscientious adolescents, as they are determined and persistent in dealing with stressful situations, are more likely to positively evaluate even the most challenging of situations, so that SRI promotes active coping and reduces emotional and avoidance coping.

Conversely, in the adult sample, the mediational role of SRI is found only in avoidance coping, while self-directed coping is directly affected by agreeableness (with a negative relation), openness, emotional stability, conscientiousness and EI (in a positive direction).

In detail, the results show the partial mediation of SRI in the relationship between conscientiousness and extraversion with avoidance coping and its full mediation in the relationship between emotional stability and EI with avoidance coping.

In our model, conscientiousness has a positive relationship with SRI, which, in turn, has a negative relationship with avoidance-oriented coping, determining the negative indirect effect of the mediation. Adults high in conscientiousness, as they are determined and persistent in dealing with situations, are inclined to also evaluate positively difficult situations, leading to less use of maladaptive coping thanks to SRI.

The same pattern is shown for extraversion; as underlined in discussing the results on adolescents, extraversion enhances a positive attitude towards uncertainty, included in the SRI construct, reducing the probability of activating maladaptive coping strategies. Similar to what we have found in adolescents, extroverted adults, without the mediation of SRI, tend to use more avoidance than active coping. Subsequently, SRI represents a protective factor in this relationship, thanks to which the energy and dynamism of the extrovert are diverted from avoidance strategies, which, in this case, concern distraction (e.g., I dedicate myself to work or other activities so as not to think about what worries me) or substance use.

Also, emotional stability and EI show the same pattern as conscientiousness and extraversion (a positive relationship with SRI, negative indirect effects). A similar explanation proposed for adolescents can be applied to adults: emotional stability, characterised by effective emotion management, enhances SRI as a resource that comprises emotional stress management [7,48] in risky and uncertain situations, so that adults with higher SRI, similarly to adolescents, are less vulnerable to emotional stress and less likely to use ineffective coping strategies. EI, characterised by the appraisal and expression of emotion in oneself and others, regulation of emotion in oneself and others and utilisation of emotions in solving problems, as seen for adolescents, improves SRI as a resource that integrates emotions and cognition in approaching risky and uncertainty situations, reducing the probability of adopting maladaptive coping strategies.

## 5. Conclusions

The results of our study suggest that SRI plays a different role in the relationships between personality, EI and coping styles strategies in adolescents and adults. After all, demographic analyses suggest that age, together with gender and culture, influence the relations between personality and coping [3]. Personality better predicted coping in the younger samples, perhaps because responses to stress are driven more strongly by temperament in younger individuals, who have had fewer opportunities to develop a range of strategies and become adept at matching them to situations [91]. Age-related personality changes, including decreases in neuroticism, extraversion and openness and increases in agreeableness and conscientiousness [41], may also have an impact. As neuroticism decreases, individuals may be less distressed and less motivated to cope, and as conscientiousness increases, they may be more likely to problem-solve, leading to less coping variability and attenuated correlations in older samples [38]. More generally, the minor role played by RI in the relationship between personality, including emotional intelligence, and coping may be related to the relationship between coping and development. We can consider coping a fundamental adaptive process that has evolutionary value in allowing people to detect, manage and learn from potentially dangerous encounters [92]. From this perspective, it is possible to hypothesise that adults have consolidated strategies less based on individual factors such as personality, emotional intelligence and SRI but more based on the interaction between these dimensions and other individual factors (emotional, motivational, cognitive and metacognitive) and environmental feedback deriving from having had, as adults, many opportunities to implement coping strategies in different contexts and domains.

## 6. Limitations

The research presented has some limitations that lead to suggestions for future works. First, the cross-sectional nature of the study does not allow us to infer causal relationships between the variables, as it cannot capture how these relationships may change over time. Future research, conducted through a longitudinal research design, will allow us to better understand the causal relationships between personality, emotional intelligence, risk intelligence and coping strategies. Second, we cannot consider the study’s sample to be representative, as we used a convenience sample, reducing the generalisability of the results to all populations. Future studies involving participants belonging to other cultural contexts could widen the knowledge of these relationships in different populations. Third, the use of self-report measures implies the possibility of some bias, such as social desirability or inaccuracies in self-perception. Including different types of measures, such as behavioural ones, may overcome this limitation in future studies. Finally, the lack of control variables, such as socioeconomic status, cultural background or life experiences, could be taken into account in further research to better isolate the unique effects of the focal constructs.

Despite these limitations, the results lead to important suggestions about future research and interventions.

To date, little research has been conducted on the construct of SRI, as it has been recently operationalised and mainly in the working and organisational domains [93]; notwithstanding, SRI can represent an important psychological resource also at the individual level, encompassing cognition and emotions that could be activated in facing uncertain and risky situations. Risk, uncertainty and frequent changes are prominent features of a risky society (as defined by Beck [2]). Thus, adolescents and adults are called to deal with an unpredictable world by developing new psychological resources to help them manage increasing uncertainty [94]. SRI can be one of these psychological resources. We have found that SRI mediates the role that personality traits and EI have in coping strategies, thus influencing the choice of effective or ineffective strategies.

These findings have important implications for both educational and therapeutic interventions. Developing and implementing SRI could benefit both adolescents and adults. Among personal resources, SRI could protect individuals from the harmful effects of stress; given that stressful events are often associated with the need for change in some salient aspect of life, people with a high level of SRI believe that change is a component of life and that this represents an opportunity for growth. Therefore, acting as a mediator, SRI, particularly in adolescence, could intervene in how personality traits affect the adoption of certain coping strategies by influencing the cognitive evaluation of events [95]. Also, for adults, SRI—regardless of its mediational role—implies fewer negative perceptions of change; these perceptions could guide their choices and planning behaviours towards using coping strategies to face or modify difficult situations rather than escape them.

## Figures and Tables

**Table 1:** (**a**). Descriptives and correlations in sample 1. (**b**). Descriptives and correlations in sample 2.

(**a**)												
	**M**	**SD**	**1**	**2**	**3**	**4**	**5**	**6**	**7**	**8**	**9**	**10**
1. Agreeableness	3.12	0.83	—									
2. Conscientiousness	3.37	0.88	0.12 **	—								
3. Emotional stability	2.69	1.03	0.21 ***	0.17 ***	—							
4. Extraversion	3.31	0.93	0.15 ***	0.06	0.11 **	—						
5. Openness	3.40	0.97	−0.05	0.06	−0.08	−0.02	—					
6. Emotional intelligence	3.71	0.43	0.10 *	0.23 ***	0.07	0.24 ***	0.19 ***	—				
7. Subjective risk intelligence	3.09	0.54	0.04	0.24 ***	0.40 ***	0.18 ***	0.11 **	0.30 ***	—			
8. Task-oriented	3.60	0.54	0.004	0.33 ***	0.07	0.01	0.11 **	0.53 ***	0.38 ***	—		
9. Emotion-oriented	3.23	0.67	−0.14	−0.18	−0.43	−0.17	0.03	0.01	−0.53	−0.01	—	
10. Avoidance-oriented	3.24	0.70	0.06	−0.03	−0.06	0.20 ***	−0.002	0.33 ***	−0.03	0.17 ***	0.23 ***	—
(**b**)												
	**M**	**SD**		**1**	**2**	**3**	**4**	**5**	**6**	**7**	**8**	**9**
1. Agreeableness	3.11	0.85		—								
2. Conscientiousness	3.81	0.87		0.01	—							
3. Emotional stability	2.85	1.05		0.25 ***	0.12 **	—						
4. Extraversion	3.30	0.88		0.08 *	0.12 **	0.08 *	—					
5. Openness	3.54	0.91		0.01	−0.02	0.001	0.02	—				
6. Emotional intelligence	3.70	0.50		0.08 *	0.22 ***	0.13 ***	0.23 ***	0.11 **	—			
7. Subjective risk intelligence	3.39	0.54		0.12 ***	0.28 ***	0.43 ***	0.22 ***	0.05	0.57 ***	—		
8. Self-directed	2.78	0.39		−0.02	0.15 ***	0.18 ***	0.04	0.12 **	0.25 ***	0.18 ***	—	
9. Avoidance	1.86	0.48		−0.04	−0.20 ***	−0.13 ***	−0.02	−0.03	−0.23 ***	−0.35 ***	0.09 *	—

Note.* *p* < 0.05, ** *p* < 0.01, *** *p* < 0.001.

**Table 2:** Subsamples’ differences for each subscale and total scale.

	Sample 1: Adolescents (N = 641)		Sample 2: Adults (N = 749)		*t*	*p*
	M	SD	M	SD		
Imaginative capability	3.39	0.71	3.55	0.55	4.58	<0.001
Positive attitude toward uncertainty	3.15	0.87	3.43	0.96	5.71	<0.001
Problem-solving self-efficacy	3.42	0.67	3.80	0.58	11.20	<0.001
Stress management	2.41	0.95	2.78	1.00	7.03	<0.001
SRIS total score	3.09	0.54	3.39	0.54	10.12	<0.001

**Table 3:** R-squared values of the model for sample 1.

		95% C.I.
Variable	R^2^	Lower
Subjective risk intelligence	0.27	0.21
Task-oriented coping	0.39	0.33
Emotion-oriented coping	0.39	0.33
Avoidance-oriented coping	0.16	0.11

**Table 4:** Effects of the personality traits and emotional intelligence on coping strategies through the mediation of SRI (sample 1).

Paths	Indirect Effects				Direct Effects		
	β	95% C.I.		*p*	β	95% C.I.	
Agreeableness–SRI–task-oriented	−0.02	−0.41	−0.03	0.025	−0.04	−1.08	0.25
Agreeableness–SRI–emotion-oriented	0.04	0.08	0.95	0.020	−0.07	−1.74	−0.09
Agreeableness–SRI–avoidance-oriented	0.01	−0.01	0.28	0.067	0.02	−0.74	1.25
Conscientiousness–SRI–task-oriented	0.03	0.12	0.50	0.002	0.19	1.26	2.52
Conscientiousness–SRI–emotion-oriented	−0.06	−1.14	−0.31	<0.001	−0.07	−1.63	−0.06
Conscientiousness–SRI–avoidance-oriented	−0.02	−0.36	−0.02	0.025	−0.09	−2.06	−0.17
Emotional stability–SRI–task-oriented	0.01	0.53	1.07	<0.001	−0.07	−1.19	−0.04
Emotional stability–SRI–emotion-oriented	−0.18	−2.31	−1.43	<0.001	−0.21	−2.94	−1.51
Emotional stability–SRI–avoidance-oriented	−0.05	−0.84	−0.15	0.005	−0.05	−1.40	0.33
Extraversion–SRI–task-oriented	0.02	0.05	0.40	0.011	−0.14	−1.88	−0.70
Extraversion–SRI–emotion-oriented	−0.05	−0.92	−0.14	0.008	−0.09	−1.79	−0.32
Extraversion–SRI–avoidance-oriented	−0.01	−0.28	−0.002	0.047	0.15	0.85	2.62
Openness–SRI–task-oriented	0.02	0.03	0.36	0.019	−0.03	−0.79	0.33
Openness–SRI–emotion-oriented	−0.04	−0.83	−0.09	0.014	0.03	−0.39	0.10
Openness–SRI–avoidance-oriented	−0.01	−0.25	0.004	0.059	−0.05	−1.45	0.23
Emotional intelligence–SRI–task-oriented	0.06	0.63	1.58	<0.001	0.45	7.77	10.46
Emotional intelligence–SRI–emotion-oriented	−0.10	−3.497	−1.639	<0.001	0.21	3.51	6.85
Emotional intelligence–SRI–avoidance-oriented	−0.03	−1.192	−0.169	0.009	0.37	7.41	11.44

**Table 5:** R-squared values of the model for sample 2.

		95% C.I.
Variables	R^2^	Lower
Subjective risk intelligence	0.48	0.42
Self-directed coping	0.11	0.07
Avoidance coping	0.14	0.10

**Table 6:** Effects of the personality traits and emotional intelligence on coping strategies through the mediation of SRI (sample 2).

Paths	Indirect Effects				Direct Effects		
	β	95% C.I.		*p*	β	95% C.I.	
Agreeableness–SRI–self-directed	0.000	−0.026	0.034	0.781	−0.072	−1.527	−0.062
Agreeableness–SRI–avoidance-directed	0.003	−0.219	0.297	0.767	−0.0047	−1.006	0.881
Conscientiousness–SRI–self-directed	−0.005	−0.179	0.067	0.375	0.0906	0.220	1.723
Conscientiousness–SRI–avoidance-directed	−0.039	−0.779	−0.237	<0.001	−0.113	−2.402	−0.544
Emotional stability–SRI–self-directed	−0.015	−0.429	0.159	0.368	0.173	0.831	2.247
Emotional stability–SRI–avoidance-directed	−0.011	−1.649	−0.813	<.001	0.019	−0.584	0.999
Extraversion–SRI–self-directed	−0.003	−0.096	0.037	0.386	−0.026	−1.090	0.537
Extraversion–SRI–avoidance-directed	−0.021	−0.514	−0.025	0.031	0.072	0.037	1.838
Openness–SRI–self-directed	0.000	−0.023	0.025	0.957	0.099	0.311	1.712
Openness–SRI–avoidance-directed	0.000	−0.213	0.226	0.957	−0.012	−1.017	0.708
Emotional intelligence–SRI–self-directed	−0.021	−1.263	0.467	0.367	0.233	2.674	5.987
Emotional intelligence–SRI–avoidance-directed	−0.160	−4.707	−2.554	<0.001	−0.033	−2.838	1.357

## Data Availability

The data are available from the corresponding author upon reasonable request.
